# Scoping review: biomarkers in chronic inducible urticaria

**DOI:** 10.3389/fimmu.2026.1909765

**Published:** 2026-09-24

**Authors:** Fauve R. van den Berge, Juliette V. L. Simons, Heike Röckmann, Martijn B. A. van Doorn

**Affiliations:** 1 Department of Dermatology, Erasmus MC, University Medical Center Rotterdam, Rotterdam, Netherlands; 2 Department of Dermatology/Allergology, University Medical Centre Utrecht, Utrecht University, Utrecht, Netherlands; 3 Centre for Human Drug Research, Leiden, Netherlands

**Keywords:** biomarker, cholinergic urticaria, CINDU, cold urticaria, delayed pressure urticaria, predictor, symptomatic dermographism

## Abstract

**Objective:**

Chronic inducible urticaria (CIndU) affects approximately 0.5% of the population and is characterized by wheals triggered by specific stimuli, such as cold or heat exposure, physical exertion, mechanical friction, vibration, or delayed pressure, distinguishing it from chronic spontaneous urticaria. Biomarkers may improve diagnosis, define endotypes, predict disease course, monitor treatment responses, and subsequently guide personalized treatment strategies. This scoping review aimed to map current evidence on diagnostic, prognostic, predictive, and monitoring or treatment-response biomarkers in CIndU, with a focus on H1-antihistamine and omalizumab therapy.

**Methods:**

This scoping review was conducted according to the Joanna Briggs Institute methodology and reported following the Preferred Reporting Items for Systematic Reviews and Meta-Analyses extension for Scoping reviews guidelines. Embase, MEDLINE, and Web of Science were searched from inception to January 29, 2026. Screening and data extraction were performed independently by two reviewers.

**Results:**

Out of 3394 screened studies, 42 studies were included, revealing a heterogeneous and exploratory evidence base with limited validation across biomarkers and CIndU subtypes. No diagnostic biomarkers were identified for several subtypes, including cold, delayed pressure, and solar urticaria. In cholinergic urticaria, sweat-related immunoglobulin E (IgE) showed moderate diagnostic potential for a sweat allergy endotype but requires validation. In symptomatic dermographism, exploratory omics-based approaches identified molecular alterations but lack clinical applicability. Across CIndU subtypes, prognostic biomarkers showed exploratory associations with disease duration and severity. In cold urticaria, provocation-based parameters were associated with disease severity and response to H1-antihistamines, while total IgE showed exploratory potential for predicting omalizumab response. Routine laboratory markers were generally not reliable for treatment response or disease monitoring in CIndU.

**Conclusion:**

Several promising exploratory biomarkers have been identified across CIndU subtypes, providing a foundation for future research. Integrative multi-omics approaches, such as those being applied in the SKINERGY CIndU pilot study within the Next Generation ImmunoDermatology consortium, hold promise for advancing biomarker discovery. External validation in large, well-characterized cohorts through international networks, such as Urticaria Centers of Reference and Excellence (UCARE), will be pivotal for clinical translation.

## 1 Introduction

Chronic urticaria (CU) is characterized by recurrent wheals and/or angioedema persisting for a minimum of six weeks (1). In chronic spontaneous urticaria (CSU), symptoms can arise without an apparent trigger, whereas in chronic inducible urticaria (CIndU), symptoms can be elicited by various specific triggers or stimuli. CIndU affects approximately 0.5% of the global population and accounts for 20–30% of all CU cases, with up to 36% of patients presenting with both CSU and CIndU (2). CIndU is differentiated by trigger or stimulus based on medical history and provocation testing (1). Symptomatic dermographism (SD) represents the most common form of CIndU, affecting 1 to 5% of the general population. It is evoked by cutaneous friction, such as scratching, rubbing, or stroking, with a whealing reaction within minutes. Conversely, in delayed pressure urticaria (DPU), a less common form of CIndU, patients develop symptoms hours after physical pressure exposure. Cold urticaria (ColdU) is a common form of CIndU and is characterized by urticaria and/or angioedema triggered by exposure to cold temperatures due to contact with cold air, water, or objects. Different subtypes of ColdU are suggested (3). Importantly, up to 70% of patients with ColdU suffer from systemic reactions, which can result in anaphylaxis due to extensive cold contact (4). Heat urticaria (HeatU), a rare form of physical urticaria, is elicited by exposure to heat on the skin, such as hot water, air, objects, and warmth of sunlight. Cholinergic urticaria (CholU) is characterized by the development of urticaria in response to sweating caused by an increase in core body temperature, for example due to physical exercise, emotional stress, or exposure to heat. Recently, different CholU subtypes differentiated by variation in specific skin manifestations have been suggested (5–7). Rarer forms of CIndU are vibratory urticaria (VibrU), solar urticaria (SolU) and aquagenic urticaria (AquaU), provoked by mechanical vibrations, ultraviolet or visible light, and contact with water respectively. Distinguishing between CU subtypes may be challenging, especially given their overlapping clinical features and the potential coexistence of multiple subtypes.

Insufficiently controlled CU can severely impact patients’ lives (8, 9). As avoidance of triggers is often infeasible, CIndU patients are confronted with challenges related to their subtype of CIndU. Management of CIndU typically begins with avoidance of the respective triggers; however, this strategy alone is often insufficient and can lead to substantial lifestyle restrictions (1). Effective treatment is therefore highly important. The current international guideline recommends CU treatment with second-generation H1-antihistamine up to fourfold standard dosage (1). In patients with insufficient disease control, first-line add-on therapy consists of omalizumab, dupilumab, and remibrutinib as parallel treatment options (1). The latter two were recently approved by both the U.S. Food and Drug Administration and the European Medicines Agency. Therapeutic strategy for all CU subtypes is the same.

Treatment efficacy has primarily been investigated in randomized controlled trials including patients mainly suffering from CSU, whereas evidence in CIndU is largely derived from real-world studies. Insufficient response to treatment with four-fold updosed H1-antihistamine in CIndU patients was reported up to 40% in a daily practice study (10). Reported ineffectiveness by CIndU patients is 30 to 70% (9, 11). Omalizumab was shown to be highly effective and safe in CIndU patients not responding to treatment with four-fold updosed H1-antihistamines, with a complete or good response in approximately 75% of patients, comparable to rates found in real-world studies investigating omalizumab effectiveness in CSU patients (12, 13).

The current treatment approach does not account for potential heterogeneity in disease characteristics between patients, even though these differences may have important clinical consequences. For example, in CSU there is a growing body of evidence for the existence of certain endotypes with variations in disease activity (14) and different treatment responses to omalizumab (15). These presumed endotypes, commonly referred to as ‘autoallergic’ and ‘autoimmune’ CSU, can be identified by measuring widely available blood-based biomarkers such as total immunoglobulin E (total IgE) and anti-thyroid peroxidase (anti-TPO) antibodies (16).

These developments can be contextualized within personalized medicine, which aims to tailor medical treatment to the individual characteristics of each patient (17). The shift from volume-based to value-based healthcare has become a guiding principle for broader health policies in the Netherlands (18) and is supported by a dedicated research agenda at European level. Consequently, several research consortia have emerged in immunodermatology (19, 20), including a Dutch nationwide Next Generation ImmunoDermatology consortium (21), which also investigates patients with CU.

Patient-specific characteristics can be considered biomarkers when they are molecular, histological, radiographical or physiological parameters and can be measured objectively and quantifiably (22, 23). Biomarkers may support the diagnosis of CIndU, help distinguish between potential subtypes within each CIndU (e.g. ‘endotypes’), aid in predicting disease course, or predict therapy response (e.g. to H1-antihistamines or omalizumab). In addition, they may be used to assess treatment response or enable better therapeutic monitoring. Accordingly, the clinical use of biomarkers may contribute to more personalized care for patients with CIndU.

Therefore, this scoping review aimed to map the currently available evidence on (exploratory) diagnostic, prognostic, predictive, treatment-response, and monitoring biomarkers in CIndU.

## 2 Methods

This scoping review was conducted systematically in accordance with the methodology outlined in the Joanna Briggs Institute (JBI) Manual for Evidence Synthesis (24), chapter “Scoping Reviews,” and reported in accordance with the Preferred Reporting Items for Systematic reviews and Meta-Analyses extension for Scoping Reviews (PRISMA-ScR) guidelines (25).

The aim of this scoping review was to address the current literature regarding biomarkers on disease stratification and/or treatment response to omalizumab or H1-antihistamines in patients with CIndU. Accordingly, diagnostic, prognostic, predictive, and monitoring or treatment-response biomarkers in CIndU were evaluated. Biomarkers were classified as follows:

- Diagnostic: biomarkers that may identify CIndU subtypes or endotypes, serve as alternatives to standard provocation testing, confirm CIndU subtypes or discriminate CIndU from CSU.
- Prognostic: biomarkers associated with the likely course of disease, including disease severity, duration, relapse, or remission, irrespective of treatment.
- Predictive: biomarkers that may predict treatment response at baseline, preferably assessed using validated patient-reported outcomes such as Urticaria Control Test (UCT), Angioedema Control Test (AECT), and Urticaria Activity Score (UAS7).
- Monitoring or treatment response: biomarkers associated with disease activity over time or treatment response during antihistamine or omalizumab therapy, preferably as defined by validated patient-reported outcomes such as UCT, AECT, and UAS7.

A broad range of biomarker modalities was thus considered, including blood- or biopsy-based measurements, *in vivo* skin tests, functional tests (such as sweat and pulmonary function tests), and provocation test outcomes (e.g. wheal size or time to wheal development). Because individual biomarkers may have multiple clinical applications, they could be included in more than one category as classified above, depending on the evidence reported in the included studies.

### 2.1 Registration

This review was registered in Open Science Framework (https://osf.io/bw8mv).

### 2.2 Search strategy

A systematic search was conducted in the Embase, MEDLINE, and Web of Science databases from inception until 29-01-2026. Search query strings are provided in **Supplementary Table 1**.

### 2.3 Selection of studies

We used the following inclusion criteria: Patients with chronic inducible urticaria (CIndU), any subtype (cold, cholinergic, symptomatic dermographism, solar, delayed pressure, heat, vibratory, and aquagenic). Measurement of biomarkers (e.g. molecular, histological, radiographic or physiological characteristics). Article written in English. In addition, for diagnostic biomarkers exploratory omics-based studies investigating molecular metabolomic, lipidomic, transcriptomic or microbiome signatures with potential diagnostic relevance were eligible, even in the absence of formal validation metrics.

Exclusion criteria were: Results not presented for a clearly defined CIndU subtype or results combining multiple CIndUs without separate analysis. No biomarker data reported in the results. For diagnostic biomarkers, studies investigating inflammatory, immunological and histological parameters or routine laboratory measurements that were not evaluated for diagnostic discrimination or clinical utility were excluded. Article not written in English. Articles regarding only urticaria other than CIndU e.g. CSU, acute urticaria, urticarial vasculitis, maculopapular cutaneous mastocytosis, systemic mastocytosis, mast cell activation syndrome, non-mast-cell-mediated angioedema, exercise-induced anaphylaxis, adult-onset Still’s disease, bullous pemphigoid (prebullous stage), Well’s syndrome, Schnitzler’s syndrome, cryopyrin-associated periodic syndromes, and bradykinin-mediated angioedema. Studies investigating contact urticaria. Non-peer-reviewed articles. Case reports, reviews, meeting abstracts or publications where no full text was available, articles in any other language than English.

Screening was performed using Covidence by two independent reviewers. In the first round, screening was based on title and abstract. If the reviewers disagreed, discrepancies were discussed and an attempt was made to reach a consensus. If a conflict could not be resolved, a third reviewer was consulted to make the final decision. In the second screening round, articles were screened based on full-text review. Conflicts were resolved as described in the first round.

### 2.4 Data extraction

A standardized data extraction sheet was created in Microsoft Excel and relevant data were extracted independently by two authors (FvdB and JS) using an initial verification stage and final extraction stage. The following data were extracted from included articles: author, year of publication, country, sample size, CIndU subtype studied, type of biomarker, sample modality, results reported, limitations.

## 3 Results

A total of 5559 records were identified. After eliminating duplicate documents, 3394 remained. Following title/abstract screening, 291 studies were left. Subsequently, after full-text screening, 42 full-text articles were included in the scoping review. The detailed flowchart of the literature search is shown in **Figure 1**. The characteristics of specific biomarkers that were considered noteworthy because of their promising results or supporting evidence, as well as biomarkers for which high-level evidence indicates no reliable association, are summarized in **Table 1**. The characteristics and outcomes of all included studies are presented in **Supplementary Tables 2**–**7**.

**Figure 1:**
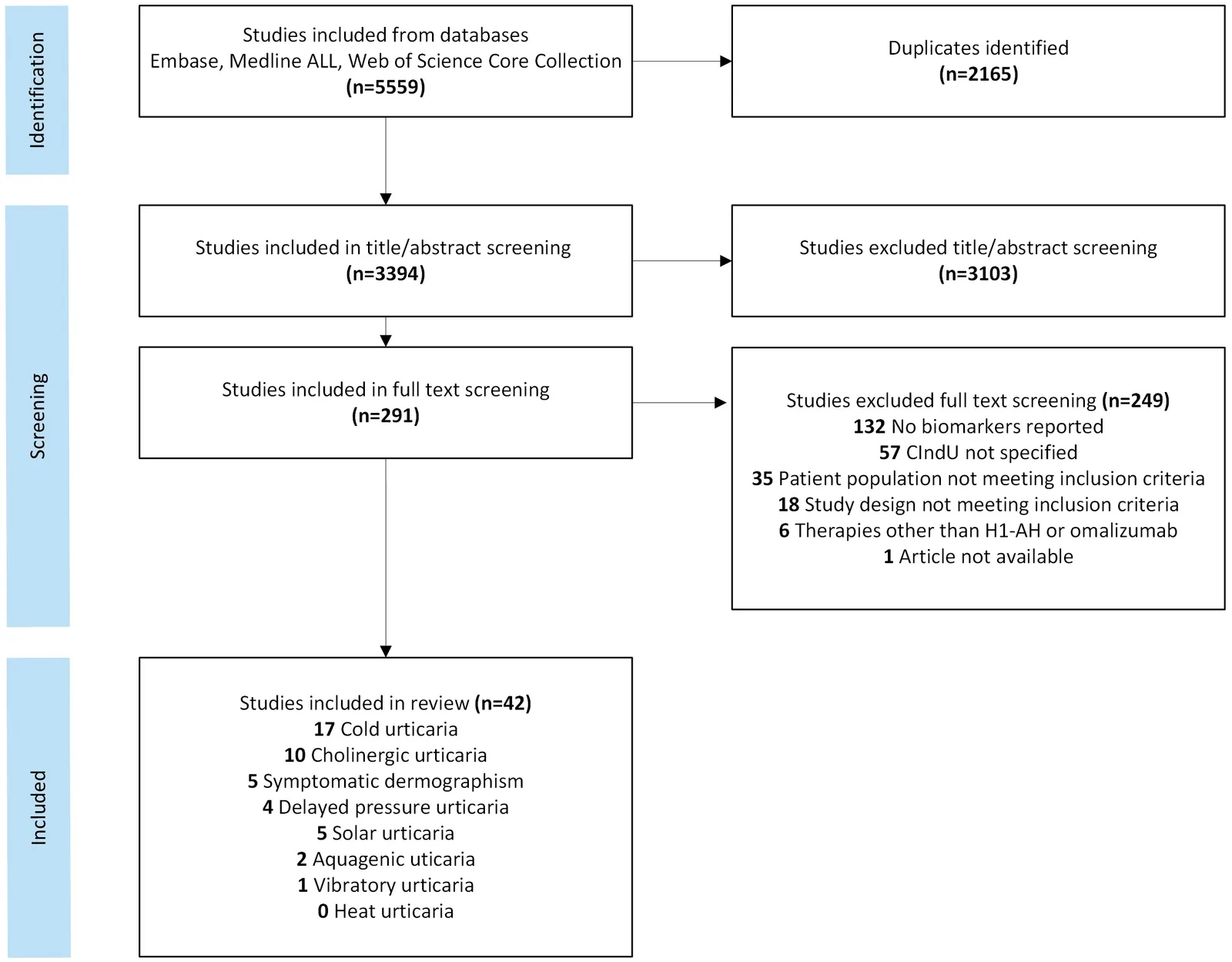
Search results during screening phase and included studies (n=42). CIndU, chronic inducible urticaria; H1-AH, H1-antihistamines. Adapted with permission from Page MJ, McKenzie JE, Bossuyt PM, Boutron I, Hoffmann TC, Mulrow CD, et al. The PRISMA 2020 statement: an updated guideline for reporting systematic reviews, licensed under CC BY 4.0, doi: 10.1136/bmj.n71.

**Table 1:** Key findings on candidate biomarkers in CIndU.

Biomarker	Specimen	Urticaria subtype	Clinical role	Main outcome	Notes/limitations	Evidence consistency	Supporting studies
Diagnostic biomarkers							
MGL_1304	Serum	CholU	Diagnosis of sweat allergy/endotype differentiation	Cutoff value ImmunoCAP(rMGL_1304): 0.671 Ua/mL, sensitivity 68.2%, specificity 95.0%.	Single study (n=42)	Low	(44)
HRN assay (semi-purified sweat antigen)	Serum	CholU	Diagnosis of sweat allergy/endotype differentiation	Sensitivity 80%, specificity 53%, positive predictive value 50%	Single study (n=28)	Low	(43)
miR-126-3p	Serum	SD	Diagnosis	Downregulated compared to controls AUC 0.769, sensitivity 68.2%, specificity 76.2%.	Single study (n=45)	Low	(52)
miR-16-5p	Serum	SD	Diagnosis	Downregulated compared to controls AUC 0.789, sensitivity 65.%, specificity 81.0%.	Single study (n=45)	Low	(52)
VEGF-A	Serum	SD	Diagnosis	Upregulated compared to controls: AUC 0.732, sensitivity 71.1%, specificity 69.1%. VEGF-A was inversely correlated with both microRNAs.	Single study (n=45)	Low	(52)
Plasma lipidomics	Plasma	SD	Diagnosis and differentiation from CSU and CSU+SD	SD vs CSU and CSU+SD: ↑ phosphatidylcholine, ↑ cholesteryl esters (signal, not discriminative) SD vs controls: ↑ phosphatidylcholine, ↑ phosphatidylethanolamines (signal, not discriminative)	Single study (n=23)	Low	(53)
Plasma metabolomics	Plasma	SD	Diagnosis and differentiation from CSU and CSU+SD	SD, CSU and CSU+SD vs controls: ↑ L-aspartate, ↓ pyruvic acid, ↓ maleic acid, ↓ N6-acetyl-L-lysine SD vs CSU and CSU+SD: ↓ 3-hydroxypicolinic acid, ↑ fatty acid related metabolites (signal, not discriminative)	Single study (n=42)	Low	(54)
Gut microbiome	Stool	SD	Diagnosis	Subdoligranulum: AUC 0.914 Ruminococcus bromii: AUC 0.921 Akkermansia: AUC 0.712 Klebsiella: AUC 0.714	Single study (n=22)	Low	(55)
Prognostic biomarkers							
Total IgE	Serum	ColdU	Differentiation in endotypes/severity	Higher in ColdU with anaphylaxis	Single study (n=92)	Low	(26)
	Serum	SolU	Differentiation in severity	Higher in moderate-severe SolU	Trend (p=0.07), conflicting findings	Low	(60, 61)
TNFα	Serum	ColdU	Differentiation in endotypes/severity	Upregulated in ColdU with anaphylaxis	Single study (n=5)	Low	(28)
Basal serum tryptase	Serum	ColdU	Differentiation in subtypes	Higher prevalence of elevated BST (>11.4ng/mL) in ColdU with anaphylaxis	Single study (n=92), trend (p=0.07)	Low	(26)
Provotella stercorea	Stool	SD	Disease duration	Lower levels may be associated with longer disease duration	Single study (n=22), trend (p=0.0596)	Low	(55)
Predictive biomarkers							
CTT	Temptest	ColdU	Response to AH and omalizumab	Higher in responders to AH. No differences between responders and non-responders to omalizumab	Single study (n=51)	Low	(31)
CSTT	Temptest	ColdU	Response to AH and omalizumab	Shorter in responders to AH. No differences between responders and non-responders to omalizumab	Single study (n=51)	Low	(31)
Total IgE	Serum	ColdU, SolarU, CholU, SD	Response to AH	No association between total IgE (>100 IU/mL) and antihistamine response	Single studies (n=23; 41)	Low	(29, 60)
	Serum	ColdU, DPU	Response to omalizumab	High in responders	Single studies (n=22; 8), no statistical analysis	Low	(30, 57)
	Serum	SolarU, CholU, DPU	Response to omalizumab	No association with response to omalizumab	Small sample size (n=6-41), results reported descriptively	Low	(49, 50, 57, 60, 62)
Monitoring or response biomarkers							
IL-6	Serum	ColdU	Monitoring treatment response	Decreased after AH treatment	Single study (n=20)	Low	(32)
IL-8	Serum	ColdU	Monitoring treatment response	Decreased after AH treatment	Single study (n=20)	Low	(32)
CTT	Temptest	ColdU	Monitoring treatment response	Association with AH response: decrease	Small sample size (n=19-45)	Low	(38–40)
CSTT	Temptest	ColdU	Monitoring treatment response	Association with AH response: increase	Small sample size (n=7-19)	Low	(40, 41)
MUD	Phototesting	SolU	Monitoring treatment response	MUD undetectable or increased after omalizumab treatment	No statistical testing	Low	(62)
MGL_1304	Serum	CholU	Monitoring/disease activity	Anti-rMGL_1304 specific IgE associated with PGA, but not UCT. Sup_MGL_1304 specific IgE associated with UASprovo driven by itch severity.	Single studies not directly comparable (n=42; n=39)	Low	(44, 51)
m227	Serum	CholU	Monitoring/disease activity	Associated with PGA, but not UCT	Single study (n=42)	Low	(44)
miR-126-3p	Serum	SD	Monitoring/disease activity	Upregulated in active disease phase compared to remission phase with no association to AH dosage	Single study: exploration (n=10) and validation cohort (n=45)	Low	(52)
miR-16-5p	Serum	SD	Monitoring/disease activity	Upregulated in active disease phase compared to remission phase with no association to AH dosage	Single study: exploration (n=10) and validation cohort (n=45)	Low	(52)
VEGF-A	Serum	SD	Monitoring/disease activity	Upregulated in active disease phase compared to remission phase with no association to AH dosage	Single study: exploration (n=10) and validation cohort (n=45)	Low	(52)

AH, antihistamine; AUC, area under the curve; CholU, cholinergic urticaria; ColdU, cold urticaria; CSU, chronic spontaneous urticaria; CSTT, critical stimulation time threshold; CTT, critical temperature threshold; DPU, delayed pressure urticaria; HC, healthy control; HRN, histamine release-neutralization; IL6, interleukine-6; IL-8, interleukine-8; miR, microRNA; MUD, Minimal urticarial dose; rMGL_1304, recombinant MGL_1304; SD, symptomatic dermographism; SolU, solar urticaria; TNFα, tumor necrosis factor α; Total IgE, total immunoglobulin E; VEGF-A, vascular endothelial growth factor A.

The characteristics of candidate biomarkers in chronic inducible urticaria that were considered noteworthy because of their promising results or remarkable negative findings. Biomarkers in orange represent biomarkers that underwent preliminary validation. Evidence consistency reflects the number of studies reporting similar findings and does not represent a formal assessment of study quality or certainty of evidence and was defined as: High, multiple prospective/validated studies or meta-analyses; Moderate, several consistent observational studies; Low, limited and/or inconsistent evidence.

### 3.1 Cold urticaria

#### 3.1.1 Diagnostic biomarkers in cold urticaria

No studies meeting the inclusion criteria were identified.

#### 3.1.2 Prognostic biomarkers in cold urticaria

Two studies included in this review describe blood-based prognostic biomarkers for identifying severe anaphylaxis associated subtypes of ColdU. A recent large cross-sectional study (n=92) found significantly higher total IgE levels in patients with cold urticaria-related anaphylaxis compared to non-anaphylactic cold urticaria patients (median 113 vs 63 IU/mL, p=0.021) (26), indicating that total IgE levels may serve as a potential biomarker for identifying ColdU patients at risk for cold-induced anaphylaxis (ColdA). This study also found a trend towards a higher prevalence of elevated basal serum tryptase (BST) (>11.4 ng/mL) in patients with ColdA compared to non-anaphylactic ColdU patients (18.2% vs 5.1%, p=0.07). However, data on event-related tryptase levels were not reported. Moreover, this study found a significantly higher prevalence of hereditary α-tryptasemia in ColdU patients independent from anaphylaxis (ColdU 10.9%, p=0.041; ColdA 15.2%, p=0.038), compared to the general population (estimated 5.7%). Moreover, one study from 2022 that included 53 children with ColdU found that elevated eosinophils were associated with an increased risk for ColdA (27).

Serum tumor necrosis factor alpha (TNF-α) level was also suggested as a potential biomarker for severe ColdU. A small study (n=5) detected TNF-α in the serum of patients with shock-like systemic reactions after cold immersion test only and not in patients with mild to no reaction (28). Considering the small sample size of this study, the authors concluded that further research was needed to confirm their findings.

#### 3.1.3 Predictive biomarkers for antihistamine and omalizumab response in cold urticaria

One study with a small sample size (n=23) investigated increased total IgE, immunoglobulin G anti-TPO and cryoproteins as predictors of antihistamine response and found no apparent association (29). However, in patients treated with omalizumab (n=22) it was reported that high total IgE at baseline was associated with a good response to omalizumab (complete response rate 22.7%, OR 12 (95% CI 1.12-128.4), p=0.01) (30), which is supported by findings of a cross-sectional study including 20 ColdU patients treated with omalizumab, reporting that all non-responders to omalizumab showed a baseline total IgE below 40 IU/mL (31).

Critical temperature threshold (CTT) and critical stimulation time threshold (CSTT) during cold provocation testing are also described as predictive biomarkers for treatment response. A large prospective study found a significant difference between responders and non-responders to antihistamines; non-responders showed a higher CTT (median 14 vs 18 °C, p=0.01) and shorter CSTT (median 3.5 vs 1 min, p=0.0004) at baseline using TempTest (31), indicating more severe disease. In contrast, no significant differences were found between responders and non-responders to omalizumab (31).

#### 3.1.4 Monitoring biomarkers in cold urticaria

Five studies (n=3–20) assessing serum histamine levels during antihistamine treatment showed inconsistent findings. Two studies found decreased histamine release or weak correlations with disease severity (32, 33), while others found no consistent association with disease activity or treatment response (34–36), suggesting that histamine levels are not reliable biomarkers for monitoring of disease severity. Similarly, evidence for autologous serum skin test (ASST) (35) and cold agglutinins (37) as disease severity monitoring biomarkers was also limited, with only small cohorts (n=3; n=35 respectively) and limited statistical testing (cryoproteins: r=−0.359, p=0.034; ASST, no statistical testing), suggesting a possible association with disease activity. Finally, peak prostaglandin D2 (PGD_2_) levels did not correlate with ColdU severity (34).

One study assessing blood-based cytokines indicated potential utility of certain biomarkers. In a prospective randomized placebo-controlled crossover study (n=20) (32), interleukin-6 (IL-6) and interleukin-8 (IL-8) levels decreased significantly after 7 days of antihistamine treatment (IL-6, 34% reduction, p=0.026; IL-8, 37% reduction, p=0.004), suggesting these cytokines may reflect treatment response.

Three studies evaluated temperature threshold measurements during antihistamine treatment. One study (n=45) found that CTT correlated with disease severity (r=0.53, p<0.05) and changes in disease activity (r=0.64, p<0.05), with greater reductions observed in patients showing marked clinical improvement (p<0.01) (38). Furthermore, two studies (both n=19) further reported significant reductions in CTT among antihistamine responders (46% reduction, p<0.001; 60% reduction, p<0.0005) (39, 40). Similarly, CSTT increased significantly following antihistamine treatment in two studies (n=7; 19) (p<0.0005; p<0.05) (40, 41). Three studies (n=6-19) reported reductions in wheal size and thickness after treatment (39, 41, 42). Together, these findings suggest that temperature threshold measurements (CTT, CSTT and wheal thickness) may be useful for assessing disease severity and activity, as well as for evaluating treatment efficacy.

### 3.2 Cholinergic urticaria

#### 3.2.1 Diagnostic biomarkers in cholinergic urticaria

Two studies evaluated blood-based biomarkers for the diagnosis of sweat allergy as a potential endotype within CholU (43, 44). A cross-sectional study published in 2012 demonstrated a positive correlation between histamine release neutralizing (HRN) assay activity and basophil histamine release in response to semi-purified sweat antigen (r=0.392, p=0.0003) (43). The HRN assay showed a sensitivity of 83%, a specificity of 53%, and a positive predictive value of 50%. More recently, a 2025 case-control study reported a strong correlation between histamine release induced by semi-purified sweat antigen and anti-recombinant MGL_1304 IgE levels (R2 = 0.648, p<0.0001) (44). Using ImmunoCAP for recombinant MGL_1304, the authors identified a diagnostic cutoff value of 0.671 UA/mL, yielding a sensitivity of 68.2% and a specificity of 95%.

#### 3.2.2 Prognostic biomarkers in cholinergic urticaria

One case-control study published in 2022 (45) and two cross-sectional studies from 2021 and 2025 (46, 47) reported exploratory biomarkers associated with disease duration in CholU. Impaired sweating responses, assessed using Minor’s iodine-starch test following pulse-controlled ergometry provocation, were associated with longer disease duration (45). Moreover, reduced acetylcholinesterase expression in both lesional and non-lesional skin was inversely correlated with disease duration (45). Positivity on the autologous sweat skin test (ASwST) in CholU showed a non-significant trend toward longer disease duration (46). Acetylcholine skin-test response was not associated with disease duration (47).

Impaired sweating was also associated with greater disease severity as measured by the Cholinergic Urticaria Severity Index (CholUSI), but not with Cholinergic Urticaria Activity Score (CholUAS7), Urticaria Activity Score after Provocation (UASProvo), Dermatology Quality of Life Index (DLQI), Cholinergic Urticaria Quality of Life Questionnaire (CholU-QoL) or UCT (46). Downregulation of acetylcholine expression was inversely correlated with CholUSI (45). However, acetylcholine skin testing was not associated with disease severity (CholUSI, CholUAS7) or quality of life (DLQI) (47).

#### 3.2.3 Predictive biomarkers for antihistamine and omalizumab response in cholinergic urticaria

A retrospective cohort study from 2024 did not identify associations between antihistamine response and increased total IgE (>100 IU/mL), elevated C-reactive protein (CRP), erythrocyte sedimentation rate (ESR), anti-TPO and anti-thyroglobulin (anti-TG) antibody positivity, or eosinopenia (29). However, these univariate analyses were based on very small sample sizes per biomarker, limiting statistical power. In addition, a cross-sectional study published in 2015 reported no apparent association between ASST or ASwST and histamine response, although formal statistical analyses were not reported (48). Similarly, univariate analysis of skin prick testing showed no association with histamine response in the 2024 cohort study and was again based on a very small sample size (29). Allergens included in the skin prick test were not specified.

Only blood-based biomarkers were evaluated for omalizumab response in CholU in the studies included in this review. Both a case series from 2019 (49) and a prospective cohort study published in 2020 (50) did not find an association between serum total IgE levels and response to omalizumab. In the prospective cohort study, linear regression analyses did not identify associations between omalizumab response and CRP, thyroid-stimulating hormone (TSH), neutrophil and basophil counts or histamine release test (HRT). Notably, in both studies, results were reported descriptively without detailed statistical analyses.

#### 3.2.4 Monitoring or response biomarkers in cholinergic urticaria

Blood-based biomarkers related to sweat allergy were evaluated for associations with disease activity and control in two cross-sectional studies published in 2020 and 2025 (44, 51) included in this review. Anti-MGL_1304 IgE was associated with physician global assessment (PGA) (44) and correlated positively with higher PCE-induced disease activity (UASProvo) scores, primarily driven by itch severity (51). Anti-m227 IgE was also associated with PGA (44). In contrast, MGL_1304 IgE levels, anti-m227 IgE levels, and the extent of histamine release in HRT using semi-purified sweat antigen (HRT(QR)) were not associated with disease control as measured by the UCT (44). Overall, these findings represent exploratory cross-sectional associations and do not establish validated monitoring biomarkers.

### 3.3 Symptomatic dermographism

#### 3.3.1 Diagnostic biomarkers in symptomatic dermographism

Circulating omics-based approaches have been explored to identify diagnostic biomarkers for SD. Three observational studies were included in this review, comprising microRNA profiling (52), lipidomics (53), and metabolomics (54).

A 2021 case-control study demonstrated that SD (n=45) is associated with downregulation of microRNAs miR-126-3p and miR-16-5p and upregulation of vascular endothelial growth factor A (VEGF-A) compared with healthy controls (n=42) (52). miR-126-3p showed an AUC of 0.769 (95% CI 0.669–0.869), with 68.2% sensitivity and 76.2% specificity, while miR-16-5p showed an AUC of 0.789 (95% CI 0.693–0.885), with 65.0% sensitivity and 81.0% specificity. A combined miR-126-3p/miR-16-5p model slightly improved discrimination (AUC 0.796, sensitivity 63.6%, specificity 83.3%). Reduced miR-126-3p expression was more pronounced in patients aged ≥45 years, and both miRNAs were lower in allergen-specific IgE-negative patients, suggesting possible disease heterogeneity. VEGF-A levels were inversely correlated with both miRNAs. VEGF-A alone showed moderate discriminatory performance (AUC 0.732, sensitivity 71.1%, specificity 69.1%), while combined biomarker panels provided only limited incremental improvement.

Another 2024 case-control study reported that untargeted metabolomic profiling distinguished CU, comprising SD (n=23), CSU (n=38), and combined CSU and SD (n=19), from healthy controls (n=82) using partial least squares discriminant analysis (R2X = 0.0859, R2Y = 0.384, Q2 = 0.189) (54). Separation was driven predominantly by alterations in energy and amino acid metabolism, including increased L-aspartate and decreased pyruvic acid, maleic acid, and N6-acetyl-L-lysine. However, substantial overlap between urticaria subtypes indicated no robust metabolic discrimination. SD exhibited subtle subtype-specific differences, including lower 3-hydroxypicolinic acid and a relative increase in fatty acid–related metabolites.

Similarly, a 2022 case-control study identified differences in plasma lipid abundance between controls (n=82), SD (n=23), CSU (n=39) and patients with combined CSU and SD (n=20) (53). However, no lipid species demonstrated clear specificity for SD compared with these groups. Lipid alterations in SD were limited and mainly involved relative enrichment of phosphatidylcholine and cholesteryl esters. Compared with healthy controls, only a small number of lipids were altered in SD, including increased phosphatidylethanolamines (36:3) and phosphatidylcholine (16:0e/16:1), with phosphatidylethanolamine (36:3) representing the only lipid consistently elevated across all urticaria subtypes. In contrast, more extensive and subtype-specific lipid changes were observed in CSU and combined CSU and SD, including enrichment of phosphatidylglycerols in CSU and increased phosphatidylethanolamines and diacylglycerols alongside reduced cholesteryl esters (20:4) in patients with combined CSU and SD (53).

In addition to circulating omics-based biomarkers, one 2021 case-control study from China explored the gut microbiome as a potential diagnostic modality (55). SD (n=22) was characterized by gut microbial dysbiosis with loss of beneficial short-chain fatty-acid-producing bacteria and expansion of opportunistic taxa compared with control (n=22). The identified diagnostic signatures were primarily depletion-based, with *Subdoligranulum* and *Ruminococcus bromii* showing the strongest discriminatory potential (AUC 0.914, 95% CI 0.834-0.995, and AUC 0.921, 95% CI 0.845-0.998, respectively).

#### 3.3.2 Prognostic biomarkers in symptomatic dermographism

Prognostic biomarkers in SD have been explored to a limited extent. One 2021 Chinese case-control study included in this review (SD n=22, controls n=22) reported exploratory findings based on gut microbiome profiling (55). Longer disease duration tended to be associated with lower *Prevotella stercorea* abundance in SD (r=−0.4176, p=0.0596).

#### 3.3.3 Predictive biomarkers for antihistamine and omalizumab response in symptomatic dermographism

Predictive biomarkers for antihistamine response in SD have been assessed in one cohort study (n=167) published in 2024 (29). Routine laboratory markers, including total IgE, CRP, ESR, anti-TPO and anti-TG positivity, and eosinophil counts, as well as skin-prick-test positivity to allergens and *Helicobacter pylori* status were not associated with H1-antihistamine response in SD (29).

#### 3.3.4 Treatment monitoring or response biomarkers in symptomatic dermographism

Molecular biomarkers for treatment monitoring in SD have been explored in one case-control study published in 2021 that was included in this review (52). Distinct expression patterns were observed for miR-126-3p, miR-16-5p, and VEGF-A between active disease and remission, suggesting potential utility as disease activity markers. However, no association was observed with varying antihistamine dose, limiting their utility for monitoring treatment response (52). Overall, these findings suggest that while molecular markers may reflect disease activity in SD, they do not reliably reflect treatment response.

### 3.4 Delayed pressure urticaria

#### 3.4.1 Diagnostic biomarkers in delayed pressure urticaria

No studies meeting the inclusion criteria were identified.

#### 3.4.2 Prognostic biomarkers in delayed pressure urticaria

One small 2011 cross-sectional study reported that elevated D-dimer levels (>0.5 µg/mL) were exclusively observed in patients with severe disease (n=3), whereas all patients with milder disease (n=5) had levels within the normal range (56). Given the small sample size and absence of longitudinal follow-up, no temporal or predictive associations could be established.

#### 3.4.3 Predictive biomarkers for omalizumab response in delayed pressure urticaria

No studies included in this review reported predictive biomarkers for antihistamine response. However, in a case series from 2022, including 14 patients with DPU, descriptive data on several blood-based biomarkers in the context of omalizumab treatment response were reported without a control group (57). Elevated inflammatory markers were variably present: ESR (>20 mm/h) in 50% of patients, CRP (>0.5 mg/dL) in 42.9%, and D-dimer (>500 ng/mL) in 3 of the 10 assessed patients. Anti-TPO levels were normal in all patients, while total IgE (>100 IU/mL) was elevated in 6 of 8 patients. These biomarker data were not statistically analyzed owing to the small sample size, and no consistent or suggestive biomarker-response patterns were identified based on the descriptive findings alone.

#### 3.4.4 Treatment monitoring or response biomarkers in delayed pressure urticaria

Two studies investigated potential monitoring or treatment-response biomarkers in DPU in the context of antihistamine therapy. In a double-blind, placebo-controlled crossover trial from 1991 (n=11), cetirizine treatment significantly reduced provocation-induced wheal size compared with both baseline (p=0.0051) and placebo (p=0.009) (58). The magnitude of this reduction was not reported numerically and was presented only graphically, limiting quantitative interpretation. Histological analysis of skin tissue obtained using the Rebuck skin window technique demonstrated a mixed inflammatory infiltrate under both cetirizine and placebo conditions. Eosinophil counts were significantly reduced during cetirizine treatment compared with placebo (p=0.0029), whereas no significant differences were observed for neutrophils, mononuclear cells, or total cell counts. In a single-group pre-post study published in 1990 (n=14) (59), histological assessment before and after three weeks of cetirizine treatment showed a significant reduction in eosinophil counts (mean 27.8 ± 14.6 vs 9.8 ± 12.2, p=0.0046), while the decrease in neutrophils was not statistically significant. Notably, the reduction in eosinophil counts reported in this study was identical to that reported in the 1991 study discussed above. Together with the highly similar cohort descriptions, this suggests overlap in reported data and was only once included in our review.

### 3.5 Solar urticaria

#### 3.5.1 Diagnostic biomarkers in solar urticaria

No studies meeting the inclusion criteria were identified.

#### 3.5.2 Prognostic biomarkers in solar urticaria

Two studies from 2024 investigated the possible association between total IgE levels and disease severity in SolU. A cohort study (n=41) found a trend toward an association (p=0.07) between high serum total IgE levels (>200 IU/L) and moderate-to-severe SolU patients with a UCT score <12, compared to patients with UCT score ≥ 12 and lower baseline total IgE levels (median 118 IU/L) (60). In addition, this study found no associations in high-affinity IgE receptor (FcϵRI) expression, antinuclear antibodies, anti-TPO, tryptase and D-dimer between patients with a UCT score <12 compared to patients with a UCT score ≥ 12 (60). However, another retrospective case-control study (n=26) did not find an association between elevated total IgE levels and more severe disease (61).

#### 3.5.3 Predictive biomarkers for antihistamine and omalizumab response in solar urticaria

Two studies (n=41 and n=6) investigated serum total IgE and did not identify associations between total IgE and response to antihistamine or omalizumab treatment (60, 62). Furthermore, associations between FcϵRI expression and disease severity or response due to treatment with antihistamines were not found in a cohort study (60). However, FcϵRI expression was suggested as a predictive biomarker for omalizumab response. A significant reduction in FcϵRI expression was found after 4 weeks of omalizumab treatment that was sustained until week 24. However, this was investigated in only 11 SolU patients that were treated with omalizumab. The same study investigated antinuclear antibodies, anti-TPO, tryptase, D-dimer and found no associations with disease severity or response to treatment with omalizumab or antihistamines (60).

A small study from 2020 (n=2) investigating photoallergen-induced basophil activation test (BAT) with *in vitro* omalizumab pre-treatment and found that basophil activation was blocked (63). The authors suggest that in SolU basophil activation is IgE-mediated and *in vitro* omalizumab BAT inhibition may predict clinical effectiveness of omalizumab in solar urticaria, although this was only investigated in 2 patients and clinical omalizumab response data was not assessed.

#### 3.5.4 Monitoring biomarkers in solar urticaria

Only plasma histamine levels were investigated as a blood-based biomarker for monitoring of SolU disease severity in a small study (n=3) from 1984 (64). After inducing tolerance with phototherapy in SolU patients, there was no longer a detectable increase in plasma histamine in two patients and only a minor increase in one patient, suggesting histamine might serve as a monitoring biomarker for disease severity.

#### 3.5.5 Biomarkers during phototesting in solar urticaria

Photoprovocation testing is the gold standard for the diagnosis of SolU. Phototest-derived parameters, such as the action spectra and minimal urticarial dose (MUD), have been investigated as potential biomarkers for disease severity and monitoring treatment response with limited evidence.

Action spectra, defined as the spectrum of wavelengths that provoke a whealing response, were determined in seven included studies from 1984 to 2024, showing inconsistent results on prevalence of positive phototests across wavelengths (UVA, UVB or visible light) and it was mostly described without evaluation of SolU endotypes or clinical consequence (60–62, 64–67). However, action spectra were evaluated as a prognostic biomarker for identification of severe SolU in one study. This retrospective case-control study (n=26) (61) reported more positive phototest results in all wavelengths (UVA, UVB, visible light) in patients in need of omalizumab treatment, indicating higher disease severity, compared to patients who were only treated with antihistamines (lower disease severity), who also had more often negative phototests, even though these differences were not statistically significant. Thus, UV action spectrum is not reliable as a prognostic biomarker.

The MUD is defined as the minimal dose in J/cm^2^ required to provoke a whealing response. One study included in this review investigated MUD for monitoring after starting treatment, showing that MUD was undetectable or increased after omalizumab (62). A case-series from 2018 showed that after omalizumab treatment 7 of 8 patients had complete response, of which three patients had no detectable MUD and 1 patient had an increased MUD (62). Although these findings may suggest a potential association between MUD and treatment response, no statistical or correlation analyses were performed to further evaluate this relationship.

## 3.6 Aquagenic, vibratory and heat urticaria

In AquaU, one study (n=9) investigated salt water (3.5% NaCl) challenge compared to tap water challenge and reported a higher diagnostic sensitivity to salt water of 77.8% vs 44.4% respectively (68), indicating that salinity is a relevant parameter for AquaU in general. The authors suggest that patients with suspected AquaU should undergo local challenge tests with both tap water and salty water, as salty water may increase the sensitivity of the test and may lower the threshold of response.

Interestingly, VibrU appears to follow an autosomal dominant inheritance pattern in certain families. Accordingly, one study performed genetic analyses (69), identifying a missense variant (p.C492Y) of adhesion G protein-coupled receptor E (ADGRE2) in patients with familial VibrU. This highlights the importance of recognizing potential hereditary forms of the disease. Notably, in non-familial VibrU patients, ADGRE2 mutations were investigated but not detected and might therefore not be relevant for nonfamilial variants (70).

In HeatU, no biomarkers were identified.

## 4 Discussion

This scoping review is the first systematic approach to identify potential biomarkers in CIndU, which may be used in clinical practice or guide exploratory in-depth profiling of CIndU in future studies. Screening and data extraction were performed independently by two researchers to minimize bias. The scoping methodology allows for mapping the currently investigated biomarker landscape of CIndU. However, the strength of the evidence is limited by the majority of studies with small sample sizes and retrospective designs. The prevalence of CIndU is generally considerably lower compared to CSU. Although studies were conducted across multiple CIndU subtypes, limited overlap in the biomarkers investigated restricts direct comparisons among subtypes and with CSU. Moreover, certain subtypes, including AquaU, VibrU, and HeatU, are exceptionally rare. As a result, these forms of CIndU remain substantially understudied, which likely contributes to the limited availability of biomarker data. Publication bias may further influence the available evidence, as exploratory studies with negative findings are less likely to be published, potentially overestimating the promise of some candidate biomarkers. Therefore, the reported biomarkers should largely be considered exploratory and hypothesis-generating rather than conclusive or ready for clinical implementation. A few biomarkers, each reported in a single study, underwent preliminary validation steps, such as MGL-1304 IgE (44) and the HRN assay using semi-purified sweat antigen (43) in CholU, and transcriptomics (52), metabolomics (54), lipidomics (53), and gut microbiome profiling (55) in SD. Nevertheless, these findings lack external validation, their clinical applicability remains uncertain, and methodological limitations hinder the interpretation and generalizability of the results. This highlights the need for well-designed, prospective studies to establish and validate clinically relevant biomarkers in CIndU.

The most extensively studied biomarkers in CSU are total IgE and anti-TPO. Type IIb ‘autoimmune’ CSU is typically associated with low total IgE, elevated anti-TPO, and poor response to omalizumab (71), suggesting their potential as predictive biomarkers. In contrast, although higher total IgE and omalizumab responsiveness have been reported in type I (71), its role in defining type I ‘autoallergic’ CSU remains debated (72). In CIndU, the role of total IgE is less well characterized and was not identified as a diagnostic biomarker in this review. Nevertheless, elevated baseline total IgE may predict omalizumab response in ColdU (30) and may be associated with more severe disease in ColdU and SolarU (26, 60, 61). These observations should be interpreted cautiously but may point to a different pattern from CSU, where total IgE appears more useful for predicting treatment response than disease course (71, 73–75). Anti-TPO was not associated with antihistamine response in most CIndU subtypes (29, 60), or with omalizumab response in SolU and DPU (57, 60), although it correlated with disease severity in SolU (60).

Interestingly, sweat allergy has been a proposed subtype of CholU (7), supported by positive sweat-induced basophil histamine release assay and ASwST in a subset of patients (46, 48, 51, 76–78). In this context, MGL_1304, a sweat-associated antigen derived from *Malassezia globosa*, represents a biologically plausible trigger (51). The present review identified anti-recombinant MGL_1304 IgE levels as an exploratory diagnostic biomarker for sweat allergy in CholU, demonstrating moderate sensitivity and high specificity (44). Functional assays using semi-purified sweat, such as the HRN assay and BAT, appear more sensitive but less specific (43, 79), reflecting broader immune reactivity without identifying the underlying antigen. However, interpretation of the BAT is limited by its evaluation in a combined CholU and atopic dermatitis cohort (79). Evidence for other proposed subtypes of CholU, such as follicular-type CholU and CholU with anhidrosis (7), remains sparse and is limited to pathomechanistic findings (44, 45, 48, 80–82). Associations between impaired sweating, reduced cholinergic marker expression, and increased disease duration and severity align with reported reduced acetylcholine receptor M3 and acetylcholinesterase expression, periglandular inflammation, and chemokine upregulation (45, 81, 82). Although sweat hypersensitivity-related tests were more frequently reported positive in non-follicular compared with follicular CholU phenotype, the mechanistic significance of this observation remains unclear (6, 46, 48, 51, 76–78).

In SD, downregulation of miR-126-3p and miR-16-5p together with VEGF-A upregulation showed moderate diagnostic performance and were associated with active disease (52). In contrast, miR-125a-5p and thymus and activation-regulated chemokine or TARC have been reported in CSU as potential diagnostic biomarkers, suggesting biological differences (52, 83). VEGF-A is involved in endothelial function and vascular permeability (84), while miR-16 has been linked to other autoimmune diseases, although its regulatory mechanisms remain unclear (85). Beyond transcriptomics, gut microbiome dysbiosis in SD showed strong discriminatory potential from healthy controls in one Chinese study (55). However, similar patterns of depletion of short-chain fatty acid-producing bacteria and expansion of opportunistic taxa, linked to heightened mast cell activation, have also been observed in CSU (86, 87). Moreover, gut microbiome studies in CSU are known to have variability due to heterogeneity in study populations, methodology, and environmental influences such as diet (88). Metabolomic and lipidomic profiling further demonstrated substantial overlap between SD and CSU, with limited discriminatory value (53, 54). Future research using standardized methodologies and integrative multi-omics approaches, including competitive endogenous RNA networks, potentially supported by artificial intelligence, may help clarify disease mechanisms and diagnostic relevance (86, 88, 89).

In ColdU, most patients experience only localized wheals and angioedema upon cold exposure. However, ColdA, a potentially life-threatening systemic reaction triggered by cooling, has been reported in over a third of patients with typical ColdU (90, 91), underscoring the need for reliable biomarkers to identify patients at risk of severe reactions. In this context, elevated total IgE, basal serum tryptase, and TNF-α have been associated with ColdA, suggesting a severe disease endotype (26, 28). Notably, elevated tryptase levels are recognized across mast cell-driven reactions, including IgE-mediated anaphylaxis and mastocytosis (92, 93), and have been associated with a more severe disease course in CSU, as reflected by greater reliance on oral corticosteroids for symptom control (94). Moreover, the potentially relevant role of TNF-α in ColdU is supported by positive TNF-α staining and strong upregulation at 30 minutes found in skin biopsies after ice cube test (95). Another study (n=20) found that TNF-α was detectable in serum of 35% of ColdU patients at baseline, however, no associations to severity were analyzed (32). In allergic patients, TNF-α was significantly elevated in patients with severe anaphylactic reactions compared with patients with moderate reactions and healthy controls (96). Additionally, in CSU, a significant positive moderate correlation was found between serum TNF-α concentrations and UAS7 scores (97), indicating a higher disease severity. However, associations with anaphylaxis or angioedema were not described. Whether comparable patterns exist in ColdU remains to be prospectively investigated. As readily accessible routine measurements, total IgE, serum tryptase, and TNF-α represent practical potential candidates for anaphylaxis risk stratification in ColdU. However, their sensitivity, specificity, and clinical relevance remain insufficiently characterized and require validation in larger, prospective studies.

Notably, previous studies have explored complement components and cryoproteins in ColdU, consistently reporting negative or clinically uninformative results (34, 36, 37, 98–116). As these were not assessed within a diagnostic framework, their routine use and further investigation appear unwarranted.

As CIndU is induced by certain triggers, provocation testing plays a central role in the diagnosis of each specific CIndU subtype. Elevated CTT and shortened CSTT as assessed by the TempTest (117) were identified as significant markers of antihistamine non-response and were associated with greater disease severity (31). However, these findings were derived from a single study with a relatively small population and no specific CTT and CSTT cutoff values were established to define thresholds predictive of treatment response. Other studies have reported a significant increase of CSTT (41), and significant decrease of CTT (40), wheal size and thickness (41, 42) after antihistamine treatment, however associations with disease severity were not analyzed. Similarly, MUD has emerged as a potential biomarker for monitoring disease activity in SolU upon treatment with omalizumab as well as phototherapy and PUVA (62, 64, 66). Availability of phototesting and to a lesser extent the TempTest may constrain future clinical application as these are largely confined to specialized centers. While the ice cube test is more widely available, it is not fully standardized and CTT and CSTT are not translatable. Consequently, there is a need for easily accessible biomarkers, for example blood-based biomarkers, that can reliably reflect disease activity and predict treatment response while minimizing the need for provocation testing.

The clinical utility of any biomarker should be carefully balanced against its cost-effectiveness. Beyond analytical validity and reliability, practical considerations such as implementation feasibility, accessibility, and costs in routine clinical practice are critical determinants of its value for patient care. We therefore consider the integration of multi-omics approaches to be a promising strategy for generating comprehensive biomarker profiles and identifying relevant disease endotypes. However, given the costs and limited accessibility associated with omics technologies, a key subsequent step will be to translate these discoveries into combinations of readily available biomarkers that can reliably capture the clinically relevant information contained within multi-omics signatures. Such biomarker panels could serve as practical and cost-effective proxies for complex molecular profiles and support diagnostic, prognostic, predictive, and treatment-monitoring applications in routine clinical practice.

In conclusion, the results of this scoping review indicate that biomarker research in CIndU remains at an exploratory stage, as current evidence is largely derived from small retrospective studies with limited external validation. Future studies should prioritize multi-center, well-characterized prospective cohorts, standardized phenotyping, harmonized outcome measures, and external validation of promising subtype-specific biomarkers to facilitate translation into clinical practice. Integrative approaches combining clinical, functional, and multi-omics biomarkers, supported by advanced analytical methods such as machine learning, may ultimately enable identification of biologically distinct CIndU endotypes and support future precision medicine strategies. In this context, ongoing initiatives such as the Dutch SKINERGY CIndU pilot study within the Next Generation ImmunoDermatology consortium, including patients with SD, ColdU, and CSU, may provide valuable opportunities for exploratory multi-omics analyses. External validation in large cohorts could potentially be facilitated through international networks such as the Urticaria Centers of Reference and Excellence (UCARE). Ultimately, implementation studies will be important to support the integration of biomarker-guided approaches into routine clinical care in an accessible and cost-effective manner.

## Data Availability

The original contributions presented in the study are included in the article/**Supplementary Material**. Further inquiries can be directed to the corresponding author.
