# Supplementary material for: Scoping review: biomarkers in chronic inducible urticaria
Source: Front Immunol. 2026 Sep 24;17:1909765. doi: 10.3389/fimmu.2026.1909765 (PMC13646115; doi:10.3389/fimmu.2026.1909765)
Supplement: Supplementary file 1 [file Supplementaryfile1.docx]

Supplementary Material

**Supplementary Table 1. Complete search strategies**

| **Database** | **Query string** | **Query 17-06-2024** | | **Query 29-01-2026** | |
| --- | --- | --- | --- | --- | --- |
|  |  | **Records** | **After duplicates removed** | **Records** | **After duplicates removed** |
| Embase | (‘inducible urticaria’/exp OR (((induc* OR aqua* OR contact* OR physical OR cholinerg* OR stress* OR sweat* OR exercis* OR exert* OR pressur* OR solar* OR thermal* OR vibrat* OR photo* OR light OR sunlight* OR temperatur* OR cold* OR heat OR warmth* OR factitia) NEAR/3 (urticaria OR hives)) OR CindU OR dermographism*):ab,ti,kw) **AND** (marker/de OR ‘biochemical marker’/de OR ‘biological marker’/de OR ‘molecular marker’/de OR ‘pharmacological biomarker’/de OR ‘immunoglobulin E’/de OR ‘blood level’/de OR ‘skin biopsy’/de OR ‘mast cell’/exp OR ‘substance P’/de OR histamine/de OR ‘Genetic marker’/de OR ‘thyroid antibody test kit’/de OR ‘feces microflora’/de OR ‘feces analysis’/exp OR ‘feces level’/de OR ‘cytokine’/exp OR ‘tissue level’/de OR (marker* OR biomarker* OR IGE OR Ig-E OR immunoglobulin-E* OR ((blood OR serum OR plasma OR tissue) NEAR/3 (level* OR concentration*)) OR biops* OR mast-cell* OR substance-p OR histamin* OR anti-TPO OR feces* OR fecal* OR faeces* OR cytokine* OR d-dimer* OR tryptase* OR CRP* OR BSE* OR ASST* OR cryoglobulin* OR cold-agglutin* OR complement* OR neurotrophic-factor* OR p-selectin* OR endotyping):ab,ti,kw) NOT ([Conference Abstract]/lim OR [Conference Review]/lim) NOT 'clinical trial'/it AND [ENGLISH]/lim | 1422 | 1416 | 1506 | 1503 |
| Medline | (exp Chronic Inducible Urticaria/ OR Cold Urticaria/ OR (((induc* OR aqua* OR contact* OR physical OR cholinerg* OR stress* OR sweat* OR exercis* OR exert* OR pressur* OR solar* OR thermal* OR vibrat* OR photo* OR light OR sunlight* OR temperatur* OR cold* OR heat OR warmth* OR factitia) ADJ3 (urticaria OR hives)) OR CindU OR dermographism*).ab,ti,kf.) **AND** (exp Biomarkers/ OR Immunoglobulin E/ OR exp Hematologic Tests/ OR Biopsy/ OR Mast Cells/ OR Substance P/ OR exp Histamine/ OR anti-thyroid autoantibodies.rs. OR exp Cytokines/ OR (marker* OR biomarker* OR IGE OR Ig-E OR immunoglobulin-E* OR ((blood OR serum OR plasma OR tissue) ADJ3 (level* OR concentration*)) OR biops* OR mast-cell* OR substance-p OR histamin* OR anti-TPO OR feces* OR fecal* OR faeces* OR cytokine* OR d-dimer* OR tryptase* OR CRP* OR BSE* OR ASST* OR cryoglobulin* OR cold-agglutin* OR complement* OR neurotrophic-factor* OR p-selectin* OR endotyping).ab,ti,kf.) NOT (congres* OR abstract*).pt. AND english.la. | 2419 | 1349 | 2581 | 1455 |
| Web of Science Core Collection | TS=(((((induc* OR aqua* OR contact* OR physical OR cholinerg* OR stress* OR sweat* OR exercis* OR exert* OR pressur* OR solar* OR thermal* OR vibrat* OR photo* OR light OR sunlight* OR temperatur* OR cold* OR heat OR warmth* OR factitia) NEAR/2 (urticaria OR hives)) OR CindU OR dermographism*)) **AND** ((marker* OR biomarker* OR IGE OR Ig-E OR immunoglobulin-E* OR ((blood OR serum OR plasma OR tissue) NEAR/2 (level* OR concentration*)) OR biops* OR mast-cell* OR substance-p OR histamin* OR anti-TPO OR feces* OR fecal* OR faeces* OR cytokine* OR d-dimer* OR tryptase* OR CRP* OR BSE* OR ASST* OR cryoglobulin* OR cold-agglutin* OR complement* OR neurotrophic-factor* OR p-selectin* OR endotyping))) NOT DT=(Meeting Abstract OR Meeting Summary) AND LA=(English) | 1384 | 415 | 1472 | 436 |
| **Total number of records** | | **5225** | **3180** | **5559** | **3394** |

**Supplementary Table 2. Biomarkers in cold urticaria**

| **Biomarker** | **Reference** | **N** | **Results** | **Interpretation** | **Evidence consistency** |
| --- | --- | --- | --- | --- | --- |
| **Diagnostic biomarkers** | | | | | |
| No diagnostic biomarkers included | | | | | |
| **Prognostic biomarkers – Blood-based** | | | | | |
| BST | Bizjak 2025 (26) | 92 | The prevalence of elevated BST levels was higher in ColdA patients compared to non-ColdA patients (18.2% vs.5.1%, p=0.065 (trend)) | Mostly negative. Higher in ColdU and CholU compared to SD | Low |
| Eosinophils | Prosty 2022 (27) | 53 | In children, elevated eosinophils were associated with cold-induced anaphylaxis (ColdA; aOR = 1.38 [95%CI: 1.04, 1.83]), which occurred in 17.3% of patients | Elevated | Low |
| Total IgE | Bizjak 2025 (26) | 92 | Total IgE higher in ColdU with anaphylaxis (n=39) than in non‑ColdA (n=53): Median 113 IU/mL vs 63 IU/mL (p=0.021), however low speciﬁcity | Increase | Low |
| TNF-α | Tillie-Leblond 1994 (28) | 5 | TNF‑α only detectable in patients who exhibited systemic (shock‑like) reactions (n=2/5). Patients with mild/no reaction and all controls did not release TNF‑α. | Increase | Low |
| **Predictive biomarkers – Blood-based** | | | | | |
| ***Antihistamine response*** | | | | | |
| Anti-TPO | Ornek Ozdemir 2024 (29) | 23 | Increased presence could not predict AH response (small sample size) | No association | Low |
| CRP |  |  |  |  |  |
| Cryoprotein |  |  |  |  |  |
| ESR |  |  |  |  |  |
| Total IgE (>100 IU/mL) |  |  |  |  |  |
| ***Omalizumab response*** | | | | | |
| Total IgE | Exposito-Serrano 2021 (30) | 22 | Higher Total IgE at baseline was associated with higher response to omalizumab [OR 12 (95% CI 1.12 - 128.4), p=0.01] | Positive association: High with response, low with non-response | Low |
|  | Gimenez- Arnau 2022 (31) | 19 | All 4 nonresponders to omalizumab had baseline IgE levels <40 Ul/ml |  |  |
| **Predictive biomarkers – Physiological/functional** | | | | | |
| ***Antihistamine response*** | | | | | |
| CTT | Gimenez- Arnau 2022 (31) | 51 | Significant difference between responders and nonresponders to AH (responders: median, 3.5; non‐responders: 1; [n1=34, n2=17], p=0.0004). Non‐responders to treatment showing higher CTT at baseline, which would be indicative of a more severe disease. | Increased in severe disease | Low |
| CSTT | Gimenez- Arnau 2022 (31) | 51 | Significant difference between responders and nonresponders to AH (responders: median, 14; non‐responders: 18; [n1=35, n2=17] 169, p=0.0108). Non‐responders to treatment showing a shorter CsTT at baseline, which would be indicative of a more severe disease. | Shorter in severe disease | Low |
| ***Omalizumab response*** | | | | | |
| CTT | Gimenez- Arnau 2022 (31) | 51 | No significant differences in CTT values at baseline between responders and non‐responders to omalizumab | No significant difference for (non)response | Low |
| CSTT | Gimenez- Arnau 2022 (31) | 51 | No significant differences in CSTT values at baseline between responders and non‐responders to omalizumab | No significant difference for (non)response | Low |
| **Monitoring biomarkers – Blood-based** | | | | | |
| ***Antihistamine response*** | | | | | |
| Cold agglutanin | Bizjak 2021 (37) | 35 | CA-positive pt had lower UCT scores = higher severity (pos; median UCT 11, IQR 7−12 vs. neg; median UCT 13, IQR 9−16; p=0.023). CA titers weakly correlated with UCT scores (severity). Titers weakly correlated with UCT scores (r =−0.359, p=0.034) | Possible association with disease severity | Low |
| Histamine | Krause 2013 (32) | 20 | Significant decrease in histamine release after treatment with AH | Inconsistent | Low |
|  | Katayama 1983 (33) | 3 | Significant decrease in histamine release after treatment with AH |  |  |
|  | Ormerod 1988 (34) | 6 | Peak histamine tended to correlate with cold sensitivity, though not statistically significant |  |  |
|  | Asero 2002 (35) | 5 | HRA results did not parallel disease activity in patients treated with AH (HRA negative in 1 patient with ongoing disease, but still positive in 1 patient in clinical remission). |  |  |
|  | Sigler 1980 (36) | 6 | No difference in histamine release in 5/6 before vs. after treatment with AH (cyproheptadine). 1/6 had reduced histamine release (67% reduction). Discussion: The antihistamine blocks the effect of histamine, not its release. Clinical improvement is due to H1 receptor blockade, not inhibition of mast-cell degranulation. |  |  |
| IL-6 | Krause 2013 (32) | 20 | IL-6 was significantly decreased (34% reduction, p=0.026) after 7 days of AH treatment (bilastine 20-80mg/day) compared to untreated patients | Decreased after H1-AH treatment | Low |
| IL-8 | Krause 2013 (32) | 20 | IL-8 was significantly decreased (37% reduction, p= =0.004) after 7 days of AH treatment (bilastine 20-80mg/day) compared to untreated patients | Decreased after H1-AH treatment | Low |
| ***Overall response/disease severity*** | | | | | |
| PGD_2_ | (34) 1998 | 6 | Increased significantly at 5 minutes after challenge (p<0.05). Levels did not correlate with severity or histamine levels. | No correlation with severity | Low |
| **Monitoring biomarkers – In vivo skin testing** | | | | | |
| ASST | (35) 2002 | 3 | ASST was found to parallel disease activity. N=3 positive ASST at baseline.  N=2 ColdU recovered and no longer showed a positive ASST  N=1 ColdU still active symptoms had a positive ASST. No p-values. | Association disease activity: Negative with response | Low |
| **Monitoring biomarkers – Physiological/functional** | | | | | |
| ***Antihistamine response*** | | | | | |
| CTT | Martinez-Escala 2015 (40) | 19 | CTT decreased significantly (p<0.0001) after 12 weeks AH (bilastine 20-80mg/day), decreased most in 80mg/day group, illustrating the beneﬁt of up-dosing (32). Mean reduction after 1 year rupatadine 20mg/day: ~60% (p<0.0005). Association between clinical improvement (and even complete remission) and a reduction in CTT. | Association with response H1-AH: decrease | Low |
|  | Mlynek 2010 (38) | 45 | Histamine and codeine-induced wheals: Correlation between CTT and wheal size (P <0.01) after AH treatment. Patients with improved disease activity had a greater reduction of CTT (p<0.01). Correlation between CTT and disease severity (r =0.53, p<0.05); milder severity = lower CTT |  |  |
|  | Gorczyza 2019 (39) | 19 | Rupatadine 20 mg significantly reduced: CTT by 46% (17.1°C → 9.6°C, p<0.001). |  |  |
| CSTT | Martinez-Escala 2015 (40) | 19 | CSTT increased after treatment with AH (rupatadine): mean improvement: +39.8% (p<0.0005). | Association with response H1-AH: increase | Low |
|  | Villas-Martinez 1992 (41) | 7 | All AH significantly increased CSTT (p<0.05). Basal: 4.4 minutes, Loratadine: 11.1, Cetirizine: 13.4, Cyproheptadine: 14.2, Ketotifen: 10.4. |  |  |
| Wheal size | Nuutinen 2007 (42) | 6 | Reduction in wheal size after AH treatment (cetirizine) (average of 50 ± 42%) | Reduction associated with response | Low |
|  | Villas-Martinez 1992 (41) | 7 | All AH significantly reduced wheal size, with maximum suppression of wheal area of 34.6% (the mean area of the wheal was reduced from 49.1 to 32.1 mm2), cetirizine 50.7% (mean area = 24.2 mm2), ketotifen 41.3% (mean area = 28.8 mm2) and cyproheptadine 63.5% with (reduction of up to 17.9 mm2). |  |  |
|  | Gorczyza 2019 (39) | 19 | Rupatadine 20 mg significantly reduced: Histamine‑induced wheals by 29% (17.8 mm → 12.8 mm, p<0.001), Codeine‑induced wheals by 30% (18.0 mm → 12.5 mm, p<0.001) |  |  |
| Wheal thickness | Nuutinen 2007 (42) | 6 | Reduction (after AH treatment cetirizine) in wheal thickness, mean change in the wheal thickness was –1.66 (visual score from 0; not elevated, to 3; clearly elevated). | Reduction associated with response | Low |

**Abbreviations**

AH, antihistamine; anti-TPO, anti-thyroid peroxidase; BST, basal serum tryptase; CA, cold agglutanin; CholU, cholinergic urticaria; ColdA, cold urticaria with anaphylaxis; ColdU, cold urticaria; CRP, C-reactive protein; CSTT, critical stimulation time threshold; CTT, critical temperature threshold; DPU, delayed pressure urticaria; ESR, erythrocyte sedimentation rate; H1-AH, Histamine 1 antihistamine; HRA, histamine release assay; IgE, total immunoglobulin E; IL6, interleukine-6; IL-8, interleukine-8; PGD2, Prostaglandin D₂; SD, symptomatic dermographism; TNFα, tumor necrosis factor α; UCT, urticaria control test.

**Legend**

Evidence consistency reflects the number of studies reporting similar findings and does not represent a formal assessment of study quality or certainty of evidence and was defined as: High, multiple prospective/validated studies or meta-analyses; Moderate, several consistent observational studies; Low, limited and/or inconsistent evidence.

**Supplementary Table 3. Biomarkers in cholinergic urticaria**

| **Biomarker** | **Reference** | **N** | **Results** | **Interpretation** | **Evidence consistency** |
| --- | --- | --- | --- | --- | --- |
| **Diagnostic biomarkers – Blood-based** | | | | | |
| MGL_1304 IgE | Takahagi 2025 (44) | 42 | HRT using semi-purified sweat antigen correlates with anti-rMGL_1304 IgE (R2=0.648, p<0.0001). Cutoff values for iCAP(rMGL_1304) for diagnosing sweat allergy was 0.671 Ua/mL (sensitivity 68.2%, specificity 95.0%). | Diagnosis sweat allergy (sens 68.2%, spec 95.0%) | Low |
| HRN assay (semi-purified sweat antigen) | Shindo 2012 (43) | 28 | HRN activity in sera of 27 patients were positively correlated with basophil HRA in response to semi-purified sweat (r=0,392, p=0.0003). Sensitivity 80%, specificity 53% and positive predictive value 50%. | Diagnosis sweat allergy (sens 80%, spec 53%, ppv 50%) | Low |
| **Prognostic biomarkers – In vivo skin testing** | | | | | |
| ASwST | Parinkarn 2025 (46) | 29 | Positive in 8/29 (27.6%). These 8 tended to have longer disease duration than ASwST negative patients 6.0 (11.0) vs. 2.0 (4.0), p=0.103. | Trend for positive ASwST patients to have longer disease duration, but not statistically significant. | Low |
| ACh skin test | Altrichter 2021 (47) | 38 | No difference between positive and negative patients in duration of disease (9.0 ± 2.13 vs. 9.0 ± 1.54, p-0.84), CholUSI (11.5 ± 3.7 vs 12.5 ± 3.8 p=0.38), CholUAS7 (47.6 ± 3.7 vs 34.2 ±8.1, p=0.22) or DLQI (9.0 ± 3.2 vs 11.0 ± 3.2, p=0.49). | Not correlated with disease duration, disease severity, or quality of life impairment. | Low |
| **Prognostic biomarkers – Skin biopsy-based** | | | | | |
| ACh expression | Wang 2022 (45) | 13 | The lower the expression of ACh in CholU in lesional and non-lesional skin, the longer (NL r=-0.69, p=0.009; L r=-0.77, p=0.002) and the more severe the disease (NL r=-0.63, p=0.02; L r=-0.58, p=0.04) as assessed by CholUSI. | Downregulation associated with longer disease duration and severe disease in patients with severe reduced sweating | Low |
| **Prognostic biomarkers – Physiological/functional** | | | | | |
| Iodine-starch test according to Minor | Wang 2022 (45) | 13 | 10/13 (77%) had reduced (7/13, 54%) or severely reduced sweating (3/13, 23%) in response to pulse-controlled ergonometry provocation. Severely reduced sweating had longer disease duration than reduced or normal sweating responders (mean±sd: 23±8.7 years vs 7.5±6.7 years; p=0.02 vs 8.0±3.4 years; p=0.05). CholUSI was highest in severely impaired sweating, followed by patients with reduced sweating, and lowest in normal sweating. Impaired sweating not linked to age at disease onset, CholUAS7 or UASProvo, DLQI, CholU-Qol, UCT, the Erlangen Atopy score, or total IgE. Total IgE was higher in severily impaired sweating compared to reduced and normal sweating (391.4±383.5 IU/ml vs 122.5±110.3 and 147±57.2 IU/ml). | Impaired sweating was associated with longer disease duration and disease severity, but not associated with disease control, activity or quality of life | Low |
| **Predictive – Blood-based** | | | | | |
| ***Antihistamine response*** | | | | | |
| Anti-TPO | Ornek Ozdemir 2024 (29) | 23 | Univariate analysis: no difference in anti-TPO positivity (>34 IU/mL) between AH refractory 1/2 (50%) and AH responsive patients 0/8, p=0.800 | No associations with antihistamine response were detected for elevated total IgE, CRP, ESR, anti-TPO and anti-TG positivity or eosinopenia. | Low |
| Anti-TG | Ornek Ozdemir 2024 (29) | 23 | Univariate analysis: no difference in anti-TG positivity (>34 IU/mL) between AH refractory 1/2 (50%) and AH responsive patients 0/6, p=0.25 |  |  |
| CRP | Ornek Ozdemir 2024 (29) | 23 | Univariate analysis: no difference in increased CRP (>5mg/L) between AH refractory 0/2 and AH responsive patients 0/7. |  |  |
| Eosinopenia | Ornek Ozdemir 2024 (29) | 23 | Univariate analysis: no difference in eosinopenia between AH refractory 1/3 (33.3%) and AH responsive patients 1/2 (50%), p=0.7 |  |  |
| ESR | Ornek Ozdemir 2024 (29) | 23 | Univariate analysis: no difference in increased ESR (>20 mm/h) between AH refractory 2/4 (50%) and AH responsive patients 0/8, p=0.091. |  |  |
| Total IgE | Ornek Ozdemir 2024 (29) | 23 | Univariate analysis: no difference in increased total IgE (>100 IU/mL) between AH refractory 1/1 (100%) and AH responsive patients 7/9 (77.8%), p=0.800. |  |  |
| ***Omalizumab response*** | | | | | |
| Basophil count | Ghazanfar 2020 (50) | 23 | Linear regression did not identify an association with response to omalizumab for basophil count. Results reported descriptively. | No association between total IgE levels, CRP, TSH, neutrophil count, basophil count or histamine release test and omalizumab response | Low |
| CRP | Ghazanfar 2020 (50) | 23 | Linear regression did not identify an association with response to omalizumab for CRP. Results reported descriptively. |  |  |
| HRT | Ghazanfar 2020 (50) | 23 | Linear regression did not identify an association with response to omalizumab for positive urticaria HR test. Results reported descriptively. |  |  |
| Neutrophil count | Ghazanfar 2020 (50) | 23 | Linear regression did not identify an association with response to omalizumab for neutrophil count. Results reported descriptively. |  |  |
| Total IgE | Ghazanfar 2020 (50) | 23 | Linear regression did not identify an association with response to omalizumab for serum total IgE levels. Results reported descriptively. |  | Low |
|  | Altrichter 2019 (49) | 16 | Total IgE levels across omalizumab response groups (complete, major, partial, and nonresponse) reported not significantly different: numerical data not provided. |  |  |
| TSH | Ghazanfar 2020 (50) | 23 | Linear regression did not identify an association with response to omalizumab for TSH. Results reported descriptively. |  | Low |
| **Predictive – In vivo skin testing** | | | | | |
| ***Antihistamine response*** | | | | | |
| ASST | Kim 2015 (48) | 18 | Histamine response not different between ASST positive or negative patients (described only). | No associations with antihistamine response were detected for ASST, ASwST, or skin prick test positivity. | Low |
| ASwST | Kim 2015 (48) | 18 | Histamine response not different between ASwST positive or negative patients (described only). |  |  |
| Skinprick test (allergens not specified) | Ornek Ozdemir 2024 (29) | 23 | Univariate analysis: no difference in skin prick test positivity between AH refractory 1/3 (33%) and AH responsive patients 0/2, p=0.6 |  |  |

**Abbreviations**
ACh, acetylcholine; AH, antihistamine; anti-rMGL_1304, anti-recombinant MGL_1304 specific IgE; anti-TG, anti-thyroglobulin; anti-TPO, anti-thyroid peroxidase; ASST, autologous serum skin test; ASwST, autologous sweat skin test; CholUAS7, Cholinergic Urticaria Activity Score for 7 days; CholU-QoL, Cholinergic Urticaria Quality of Life Questionnaire; CholUSI, Cholinergic Urticaria Severity Index; CRP, C-reactive protein; DLQI, Dermatology Life Quality Index; ESR, erythrocyte sedimentation rate; HRN assay, histamine release neutralizing assay; HRT, histamine release test; sd, standard deviation; total IgE, total immunoglobulin E; TSH, thyroid stimulating hormone; UASProvo, Urticaria Activity Score during Provocation testing; UCT, Urticaria Control Test.

**Legend**

Evidence consistency reflects the number of studies reporting similar findings and does not represent a formal assessment of study quality or certainty of evidence and was defined as: High, multiple prospective/validated studies or meta-analyses; Moderate, several consistent observational studies; Low, limited and/or inconsistent evidence.

**Supplementary Table 4. Biomarkers in symptomatic dermographism**

| **Biomarker** | | **Reference** | **N** | **Results** | **Interpretation** | **Evidence consistency** |
| --- | --- | --- | --- | --- | --- | --- |
| **Diagnostic biomarkers – Blood-based** | | | | | | |
| Metabolomics | Jian 2024 (54) | | 42 | Untargeted metabolomic profiling demonstrated separation between chronic urticaria phenotypes (SD, CSU, combined CSU and SD) and healthy controls on PLS-DA analysis (R2X=0.0859, R2Y=0.384, Q2=0.189) characterized by increased L-aspartate and decreased N6-acetyl-L-lysine, maleic acid and pyruvic acid. However, there was substantial overlap between urticaria subtypes with no robust discrimination (R2X=0.117, R2Y=0.398, Q2=20.0931). Supplementary data showed that SD compared to controls was characterized predominantly decreased metabolites involved in energy and amino acid metabolism (e.g. pyruvic acid, maleic acid, N‑acetylornithine, 3‑phosphoglyceric acid and N6‑acetyl‑L‑lysine) and increased L‑aspartate, L‑arginine, and arachidonic acid. Compared with CSU, SD showed increased fatty acid–related metabolites (e.g. oleic acid, myristic acid) and decreased 3‑hydroxypicolinic acid and N‑acetylornithine.  Compared with combined CSU and SD, SD showed lower levels of 3‑hydroxypicolinic acid and energy-/amino acid–related metabolites (e.g. 3‑phosphoglyceric acid), and higher levels of fatty acid–related metabolites (e.g. hexadecanedioic acid) and methylxanthines (paraxanthine, 1‑methylxanthine). | Distinct metabolomic profile for chronic urticaria (comprising SD, CSU, combined CSU and SD) compared to controls, driven by alterations in energy and amino acid metabolism (e.g. ↑ L‑aspartate, ↓ pyruvic acid, ↓ maleic acid, ↓ N6‑acetyl‑L‑lysine).  However, no robust metabolomic discrimination between SD, CSU, and combined CSU and SD due to substantial overlap.  SD showed subtle subtype-specific metabolic differences, including lower 3‑hydroxypicolinic acid and a relative shift toward relatively increased fatty acid–related metabolites. | Low |
| miR-126-3p | Gao 2021 (52) | | 45 | Downregulated in SD vs controls (0.036 [0.016; 0.062] vs 0.087 [0.047; 0.258], p<0.0001). AUC 0.769 (95% CI, 0.669–0.869, p<0.0001), sens 68.2%, spec 76.2%. miR-126-3p + miR-16-5p: AUC 0.796 (95% CI, 0.702–0.889, p<0.0001), sens 63.6%, spec 83.3%.  Lower in patients ≥45 years (0.022 IQR 0.011; 0.041 vs 0.043 (IQR 0.027; 0.132, p=0.008) and allergen-specific IgE negative patients (0.060 IQR 0.026; 0.285 vs 0.031 IQR 0.012; 0.044, p=0.020). | Associated downregulation of: - miR-126-3p: AUC 0.769, sensitivity 68.2%, specificity 76.2% - miR-16-5p: AUC 0.789, sensitivity 65.0%, specificity 81.0%  - Combination of miR-126-3p and miR-16-5p: AUC 0.796, sensitivity 63.6%, specificity 83.3%  Reduced miR-126-3p was more pronounced in patients ≥45 years, and both microRNAs in allergen-specific IgE-negative patients, suggesting disease heterogeneity.  Associated upregulation of VEGF-A: AUC 0.732, sensitivity 71.1%, specificity 69.1%  VEGF-A was inversely correlated with both miRNAs.  Combined models, including triple-marker panels, provided only marginal additional benefit with trade-offs between sensitivity and specificity. | Low |
| miR-16-5p | Gao 2021 (52) | | 45 | Downregulated in SD vs controls (0.128 [0.043; 0.303] vs 0.367 [0.218; 1.000], p<0.0001). AUC 0.789 (95% CI, 0.693–0.885, p<0.0001), sens 65.%, spec 81.0%. miR-126-3p + miR-16-5p AUC: 0.796 (95% CI, 0.702–0.889, p<0.0001), sens 63.6%, spec 83.3%.  Lower in allergen-specific IgE negative patients (0.381 IQR 0.129; 0.961 vs 0.090 IQR 0.027 0.188, p =0.0004). |  |  |
| VEGF-A | Gao 2021 (52) | | 45 | Inversely correlated with miR-126-3p (ρ=−0.587, p<.0001) and miR-16-5p (ρ=−0.458, p =0.0018). AUC 0.732 (95% CI, 0.626–0.838, p=0.0002), sens 71.1%, spec 69.1%. VEGF-A + miR-126-3p AUC: 0.767 (95% CI, 0.677–0.866, p<0.0001), sens 75.0%, spec 71.4%. VEGF-A + miR-16-5p AUC: 0.791 (95% CI, 0.694–0.887, p<0.0001), sens 68.2%, spec 81.0%. VEGF-A + miR-16-5p + miR -126-3p AUC: 0.775 (95% CI, 0.677–0.873, p<0.0001), sens 77.3%, spec 73.8%. |  |  |
| Plasma lipidomics | Li 2022 (53) | | 23 | SD was primarily characterized by increased phosphatidylcholines and cholesteryl esters, with phosphatidlethanolamine (36:3) and phosphatidylcholine (16:0e/16:1) elevated compared with healthy controls. Notably, phosphatidlethanolamine (36:3) was the only lipid consistently altered across SD, CSU and combined CSU and SD. In contrast, CSU showed broader increase in phosphatidylglycerol species. Compared with combined CSU and SD, SD lacked the marked elevation of diacylglycerols (18:0/18:1,18:1/18:1, 20:1/18:1), which were specific to the combined phenotype, while cholesteryl ester (20:4) was reduced in combined CSU and SD. | No lipid showed clear specificity for SD versus controls; SD exhibited minimal lipidomic remodeling, with phosphatidylcholines and cholesteryl ester associated changes and phosphatidlethanolamine (36:3) as a shared marker across SD, CSU and combined CSU and SD. diacylglycerol elevation and reduced cholesteryl ester (20:4) characterized combined CSU and SD but not SD alone, while phosphatidylglycerol and broader phosphatidlethanolamine changes were driven by CSU. | Low |
| **Diagnostic biomarkers – Stool-based** | | | | | | |
| Gut microbiome | Liu 2021 (55) | | 22 | SD patients showed reduced alpha diversity and distinct beta diversity vs controls. Predominant depletion of short-chain fatty acid-producing taxa (e.g. *Subdoligranulum*, *Ruminococcus bromii*, Ruminococcaceae, Lachnospiraceae) and increase in Enterobacteriaceae-related taxa (incl. *Klebsiella*). LEfSe identified discriminatory taxa. ROC analysis: *Subdoligranulum* (AUC 0.914), *Ruminococcus bromii* (AUC 0.921), *Akkermansia* (AUC 0.712), *Klebsiella* (AUC 0.714). Functional prediction showed ↓ butyrate, pyruvate, and histidine metabolism. | Associated with gut microbial dysbiosis with loss of beneficial short-chain fatty acid-producing bacteria and expansion of opportunistic taxa. Diagnostic signatures are primarily depletion-based, with *Subdoligranulum* and *Ruminococcus bromii* showing the strongest discriminatory potential (AUC 0.914 and 0.921 respectively). | Low |
| **Predictive biomarkers – Blood-based** | | | | | | |
| ***Antihistamine response*** | | | | | | |
| Anti-TPO | Ornek Ozdemir 2024 (29) | | 167 | Univariate analysis: no difference in anti-TPO positivity (>34 IU/mL) between AH refractory 3/18 (16.7%) and AH responsive patients 6/81 (7.4%), p=0.207 | Increased CRP, ESR, anti-TPO and anti-TG positivity, and eosinopenia could not predict H1 antihistamine treatment response, but univariate analyses are based on small sample sizes. | Low |
| Anti-TG | Ornek Ozdemir 2024 (29) | | 167 | Univariate analysis: no difference in anti-TG positivity (>34 IU/mL) between AH refractory 3/15 (20%) and AH responsive patients 5/67 (7.5%), p=0.157 |  |  |
| CRP | Ornek Ozdemir 2024 (29) | | 167 | Univariate analysis: no difference in increased CRP (>5mg/L) between AH refractory 4/17 (23,5%) and AH responsive patients 12/76, p=0.327. |  |  |
| Eosinopenia | Ornek Ozdemir 2024 (29) | | 167 | Univariate analysis: no difference in eosinopenia between AH refractory 3.12 (25%) and AH responsive patients 3.43 (7%), p=0.11. |  |  |
| ESR | Ornek Ozdemir 2024 (29) | | 167 | Univariate analysis: no difference in increased ESR (>20 mm/h) between AH refractory 4/15 (26.7%) and AH responsive patients 16/72 (22.2%), p=0.47 |  |  |
| Skinprick test (not specified) | Ornek Ozdemir 2024 (29) | | 36 | SPT (+) was not related to AH response (2/7 (28%) in refracotry vs 14/29 (48.3%) in responsive patients, p=0.306 |  |  |
| Total IgE | Ornek Ozdemir 2024 (29) | | 167 | Univariate analysis: no difference in increased total IgE levels (100 IU/mL) between AH refractory 14/15 (93.3%) and AH responsive patients 64/84 (76.2%), p=0.12 or mean value 191 vs 160, p=0.322. |  |  |
| **Predictive biomarkers – Stool-based** | | | | | | |
| ***Antihistamine response*** | | | | | | |
| Helicobacter Pylori | Ornek Ozdemir 2024 (29) | | 56 | Positivity was not predictive of H1 antihistamine refractoriness (7/9, 77.8% refractory vs 23/47 (48.9%) responsive patients, p=0.109). | Helicobacter Pylori positivity not associated with antihistamine response, but the sample size was very low for AH refractory patients | Low |
| **Monitoring or response biomarkers – Blood-based** | | | | | | |
| miR-126-3p | Gao 2021 (52) | | 10 | Upregulated in active disease phase compared to remission phase (0.114 IQR 0.027 vs 0.016 IQR 0.008;0.037; 0.229, p=0.0039). | May function as disease-activity biomarkers (active vs. remission phases) with no association to antihistamine dose, indicating no treatment-response utility. | Low |
|  | Gao 2021 (52) | | 45 | No difference in expression among completely controlled patients across antihistamine dose groups. |  |  |
| miR-16-5p | Gao 2021 (52) | | 10 | Upregulated in active disease phase compared to remission phase (1.68 IQR 0.530 vs 0.078 IQR 0.020; 0.136; 3.23, p=0.0020). |  |  |
|  | Gao 2021 (52) | | 45 | No difference in expression among completely controlled patients across antihistamine dose groups. |  |  |
| VEGF-A | Gao 2021 (52) | | 10 | Decreased in the remission versus active phase (378.8 IQR 354.4; 464.9 vs 451.8 IQR 440.2; 590.5, p=0.0059). |  |  |
|  | Gao 2021 (52) | | 45 | No difference in expression among completely controlled patients across antihistamine dose groups |  |  |

**Abbreviations**

Anti-TG, anti-thyroidglobulin; anti-TPO, anti-thyroid peroxidase; AUC, area under the curve; CRP, C-reactive protein; CSU, chronic spontaneous urticaria; ESR, erythrocyte sedimentation rate; IgE, total immunoglobulin E; IQR, interquartile range; PLS-DA, partial least-squares discriminant analysis; ROC analysis, receiver operating characteristic analysis; SD, symptomatic dermographism; Total VEGF-A, vascular endothelial growth factor A.

**Legend**

Evidence consistency reflects the number of studies reporting similar findings and does not represent a formal assessment of study quality or certainty of evidence and was defined as: High, multiple prospective/validated studies or meta-analyses; Moderate, several consistent observational studies; Low, limited and/or inconsistent evidence.

**Supplementary Table 5. Biomarkers in delayed pressure urticaria**

| **Biomarker** | **Reference** | **N** | **Results** | **Interpretation** | **Evidence consistency** |
| --- | --- | --- | --- | --- | --- |
| **Diagnostic biomarkers** | | | | | |
| No diagnostic biomarkers reported | | | | | |
| **Prognostic biomarkers – Blood-based** | | | | | |
| D-dimer | Kasperska-Zajac 2011 (56) | 8 | Elevated in 3/3 severe patients. Normal in 5/5 milder patients. | May indicate patients with severe disease | Low |
| **Predictive biomarkers – Blood-based** | | | | | |
| ***Omalizumab response*** | | | | | |
| Anti-TPO | Veleiro-Perez 2022 (57) | 14 | Normal in all patients. Based on descriptive results, no recognizable biomarker pattern emerged related to treatment response. | No association with omalizumab response | Low |
| CRP | Veleiro-Perez 2022 (57) | 14 | Elevated (>0.5 mg/dL) in 42.85%. Based on descriptive results, no recognizable biomarker pattern emerged related to treatment response. |  |  |
| D-dimer | Veleiro-Perez 2022 (57) | 14 | Elevated (>500.0 ng/mL) in 3/10. Based on descriptive results, no recognizable biomarker pattern emerged related to treatment response. |  |  |
| ESR | Veleiro-Perez 2022 (57) | 14 | Elevated (>20.0 mm/h) in 50%. Based on descriptive results, no recognizable biomarker pattern emerged related to treatment response. |  |  |
| Total IgE | Veleiro-Perez 2022 (57) | 14 | Elevated (>100 IU/mL) in 6/8 patients. Based on descriptive results, no recognizable biomarker pattern emerged related to treatment response. |  |  |
| **Monitoring biomarkers – In vivo skin testing** | | | | | |
| Pressure induces whealsize | Kontou-Fili 1991 (58) | 11 | Reduced after cetirizine treatment compared to before treatment (p=0.0051) and placebo (p=0.009), sizes only reported graphically | Reduced after, cetirizine treatment | Low |
| Skin window (cell counts) | Kontou-Fili 1991 (58) | 11 | Reduced eosinophil counts (p=0.0029) after cetirizine treatment compared to placebo treatment. No differences in neutrophils, mononuclear or total cell counts. | Reduced after cetirizine treatment | Low |
| **Monitoring biomarkers – Skin biopsy-based** | | | | | |
| Eosinophil count | Kontou-Fili 1990 (59) | 14 | Reduced after cetirizine treatment: 27.8 sd 14.6 vs 9.8 sd 12.2, p=0.0046), | Reduced after cetirizine treatment | Low |
| Neutrophil count | Kontou-Fili 1990 (59) | 14 | Trend of reduction after cetirizine treatment: 22 sd 17.5 vs 12.2 sd 13.4, p=0.0624 | Trend of reduction after cetirizine treatment | Low |

**Abbreviations**

Anti‑TPO, anti‑thyroid peroxidase antibodies; CRP, C-reactive protein; ESR, erythrocyte sedimentation rate; sd, standard deviation; Total IgE, total immunoglobulin E.

**Legend**

Evidence consistency reflects the number of studies reporting similar findings and does not represent a formal assessment of study quality or certainty of evidence and was defined as: High, multiple prospective/validated studies or meta-analyses; Moderate, several consistent observational studies; Low, limited and/or inconsistent evidence.

**Supplementary Table 6. Biomarkers in solar urticaria**

| **Biomarker** | **Reference** | **N** | **Results** | **Interpretation** | **Evidence consistency** |
| --- | --- | --- | --- | --- | --- |
| **Diagnostic biomarkers** | | | | | |
| No diagnostic biomarkers reported | | | | | |
| **Prognostic biomarkers – Blood-based** | | | | | |
| ANA | Pesque 2024 (60) | 41 | Patients with UCT <12 at baseline (moderate-severe disease) (N=16/41) VS patients with UCT>12 at baseline (mild disease) (n=25/41): Positive in 3 (19%) vs 1 (4%), p=0.12 | Not predictive of disease severity | Low |
| Anti‑TPO | Pesque 2024 (60) | 41 | Patients with UCT <12 at baseline (moderate-severe disease) (N=16/41) VS patients with UCT>12 at baseline (mild disease) (n=25/41): Positive in 3 (19%) vs 1 (4%), p=0.12 | Not predictive of disease severity | Low |
| D‑dimer | Pesque 2024 (60) | 41 | Patients with UCT <12 at baseline (moderate-severe disease) (N=16/41) VS patients with UCT>12 at baseline (mild disease) (n=25/41): Median 265 (430–190) 230 (340–190) mg/dL, p=0.48 | Not predictive of disease severity | Low |
| FcεRI expression (basophils, MFI) | Pesque 2024 (60) | 41 | Patients with UCT <12 at baseline (moderate-severe disease) (N=16/41) VS patients with UCT>12 at baseline (mild disease) (n=25/41): Median 12,833 (17,230–5,187) vs 10,906.5 (17,385–7,228), p=0.80 | Not predictive of disease severity | Low |
| Tryptase | Pesque 2024 (60) | 41 | Patients with UCT <12 at baseline (moderate-severe disease) (N=16/41) VS patients with UCT>12 at baseline (mild disease) (n=25/41): Median 4.8 (5.9–3.2) vs 3.6 (5.2–3.0) ng/mL, p=0.38 | Not predictive of disease severity | Low |
| Total IgE | Casanova-Esquembre 2024 (61) | 26 | 77% (10/13) of the patients in the omalizumab group (higher severity) showed abnormal IgE levels with mean levels of 350 IU/mL vs 53.8% (7/13) with mean levels of 197 IU/mL in the AH group, p=0.142 | Association between higher severity and higher IgE (NS) | Low |
|  | Pesque 2024 (60) | 41 | Patients with UCT <12 at baseline (moderate-severe disease) (N=16/41) VS patients with UCT>12 at baseline (mild disease) (n=25/41): Median 200 (636–62.5) vs 118 (279–35), p=0.07 (trend) |  |  |
| **Predictive biomarkers – Blood-based** | | | | | |
| ***Antihistamines*** | | | | | |
| ANA | Pesque 2024 (60) | 41 | No significant differences between patient with or without remission (timepoint unclear) due to treatment with AH | Not predictive for response to AH | Low |
| Anti‑TPO | Pesque 2024 (60) | 41 | No significant differences between patient with or without remission (timepoint unclear) due to treatment with AH |  |  |
| D‑dimer | Pesque 2024 (60) | 41 | No significant differences between patient with or without remission (timepoint unclear) due to treatment with AH |  |  |
| FcεRI expression (basophils, MFI) | Pesque 2024 (60) | 41 | No significant differences between patient with or without remission (timepoint unclear) due to treatment with AH |  |  |
| Tryptase | Pesque 2024 (60) | 41 | No significant differences between patient with or without remission (timepoint unclear) due to treatment with AH |  |  |
| Total IgE | Pesque 2024 (60) | 41 | No significant differences between patient with or without remission (timepoint unclear) due to treatment with AH or omalizumab |  |  |
| ***Omalizumab*** | | | | | |
| ANA | Pesque 2024 (60) | 11 | No significant differences between patient with or without remission (timepoint unclear) due to treatment with omalizumab | Not predictive for response to omalizumab | Low |
| Anti‑TPO | Pesque 2024 (60) | 11 | No significant differences between patient with or without remission (timepoint unclear) due to treatment with omalizumab | Not predictive for response to omalizumab | Low |
| Basophil CD203c expression / BAT | Oda 2020 (63) | 2 | During BAT, pretreatment of basophils with omalizumab blocked IgE binding (passive sensitization) and thereby blocked serum photo-allergen-induced basophil activation. Conclusion: BAT may predict effectiveness of omalizumab | BAT may predict response | Low |
| D‑dimer | Pesque 2024 (60) | 11 | No significant differences between patient with or without remission (timepoint unclear) due to treatment with omalizumab | Not predictive for response to omalizumab |  |
| FcεRI expression | Pesque 2024 (60) | 11 | Clear cut reduction of FcεRI expression after omalizumab treatment (no p-values). FcεRI identified as a possible predictor of omalizumab response. FcεRI expression (basophils, MFI) Median 10,287 (IQR 15,060–4,510). After 4 weeks of omalizumab treatment: clearcut decrease in FcεRI levels, stable up to 24 weeks of treatment. At 24 weeks of treatment 11/11 have UCT>12, and 7/11 even complete response (UCT16) | Reduction after omalizumab | Low |
| Tryptase | Pesque 2024 (60) | 11 | No significant differences between patient with or without remission (timepoint unclear) due to treatment with omalizumab | Not predictive for response to omalizumab | Low |
| Total IgE | Morgado-Carrasco 2018 (62) | 6 | Elevated in all tested patients (6/6, median 347 IU/L, range 136-2874 IU/L and reference level < 100 IU/L), but not predictive of omalizumab response.  In our literature review, 76.9% (10/13) of the responders with available data presented elevated IgE serum levels vs 50% (2/4) of nonresponders. | Not predictive of omalizumab response | Low |
|  | Pesque 2024 (60) | 41 | No significant differences between patient with or without remission (timepoint?) due to treatment with omalizumab or AH |  |  |
| **Predictive biomarkers – In vivo skin testing** | | | | | |
| Action spectrum | Casanova-Esquembre 2024 (61) | 26 | More positive phototest results in all wavelengths in patients in need of treatment with omalizumab (higher severity, n=13) vs AH (lower severity, n=13): 23.1% vs 7.7%. AH group had more patients with negative phototests (38.5% vs 7.7%) | Association more photosensitivity correlated with higher disease severity | Low |
| **Monitoring biomarkers – Blood-based** | | | | | |
| Histamine | (64) 1984 | 3 | After inducing tolerance, in 2/3 no detectable rise in plasma histamine, in 1/3 minor rise to ~6.5 ng/mL at 20 min, coinciding with sparse micropapular wealing, not the confluent urticarial reaction seen pre‑tolerance. | Decrease histamine after tolerance | Low |
| **Monitoring biomarkers – Physiological/functional** | | | | | |
| ***Omalizumab*** | | | | | |
| MUD | (62) 2018 | 8 | After omalizumab treatment (median time on omalizumab 16 months (range, 3-­60), and maintenance dosage was 300 mg every 4 weeks in 6 patients and 450 mg every 4 weeks in the other 2.): 89% (7/8) achieved complete response and BAAHNL.  3/8 (37.5% had normal phototesting and MUD increased in 1/8 (12.5%) | Decrease after omalizumab | Low |

**Abbreviations**

AH, antihistamine; ANA, antinuclear antibodies; anti‑TPO, anti‑thyroid peroxidase antibodies; BAAHNL, being asymptomatic and able to have a “normal life”; BAT, basophil activation test; FcεRI, high-affinity IgE receptor; MUD, Minimal urticarial dose; Total IgE, total immunoglobulin E; UCT, urticaria control test.

**Legend**

Evidence consistency reflects the number of studies reporting similar findings and does not represent a formal assessment of study quality or certainty of evidence and was defined as: High, multiple prospective/validated studies or meta-analyses; Moderate, several consistent observational studies; Low, limited and/or inconsistent evidence.

**Supplementary Table 7. Biomarkers in aquagenic, vibratory and heat urticaria**

| **Biomarker** | **Reference** | **N** | **Results** | **Interpretation** | **Evidence consistency** |
| --- | --- | --- | --- | --- | --- |
| **Aquagenic urticaria** | | | | | |
| **Diagnostic biomarkers – Physiological/functional** | | | | | |
| (Salt)water provocation test | Gallo 2024 (68) | 9 | Salt-water (3.5% NaCl) provocation test: 7/9 patients showed a reaction with wheals. 2/9 showed patches of faint itchy erythema.  Tap water test: 4/9 patients had a positive reaction with wheals, 1/9 had patches of faint itchy erythema, 4/9 were negative. The 4 negative tap water-soaked pads tested patients had positive full body tap water tests.  Conclusion: Salt-water challenge tests seems to have a higher sensitivity than the tap water challenge test when using soaked pads on specific areas in AquaU reporting symptoms triggered by salt water. | Salt-water challenge tests seems to have a higher sensitivity than the tap water challenge test | Low |
| **Predictive biomarkers – Blood-based** | | | | | |
| IgE | Gallo 2024 (68) | 9 | IgE levels measured and H1 antihistamine response reported, seems inconsistent, however not statistically compared.  Total IgE, response: 31.6, partial; 130.0, good; 89.0, poor; 47.8, partial; 300.0, good; 150.0, partial; 70.9, poor; 45.6, no treatment; 176.0, partial. | Unclear | Low |
| **Vibratory urticaria** | | | | | |
| **Diagnostic biomarkers – Blood-based** | | | | | |
| ADGRE2 mutation | Zhao 2019 (70) | 3 | Not found in nonfamilial forms of VibU | ADGRE2 mutation was identified as basis of autosomal dominant (familial) VibU, however, not in the nonfamilial variant | Low |
|  | Boyden 2016 (69) | Unclear | A missense variant in ADGRE2 was identified as basis of autosomal dominant (familial) VibU |  |  |
| **Heat urticaria** | | | | | |
| No biomarkers reported | | | | | |

**Abbreviations**

ADGRE2, adhesion G protein-coupled receptor E; AquaU, aquagenic urticaria; Total IgE, total immunoglobulin E; VibU, vibratory urticaria

**Legend**

Evidence consistency reflects the number of studies reporting similar findings and does not represent a formal assessment of study quality or certainty of evidence and was defined as: High, multiple prospective/validated studies or meta-analyses; Moderate, several consistent observational studies; Low, limited and/or inconsistent evidence.
